# The Role of the Natural Antioxidant Mechanism in Sperm Cells

**DOI:** 10.1007/s43032-021-00795-w

**Published:** 2021-11-29

**Authors:** Alicja Kowalczyk

**Affiliations:** Department of Environment Hygiene, and Animal Welfare, Wrocław University Of Environmental and Life Sciences, Chełmońskiego 38C, Wroclaw, Poland

**Keywords:** Antioxidants, Cell, Mechanism, Spermatozoa, Reproduction

## Abstract

Molecular studies of the causes of male infertility revealed a significant contribution of oxidative stress. When excessive amounts of reactive oxygen species (ROS) are produced or antioxidant activity fails, the equilibrium between oxidation and reduction is disrupted, causing oxidative stress (OS). High levels of ROS can have an adverse effect on sperm function through the initiation of DNA damage, lipid peroxidation, loss of membrane integrity and increased permeability, inactivation of cellular enzymes, and cell apoptosis. In addition to endogenous factors such as immature sperm, leukocytes, and varicocele, potential causes of excessive ROS can also be found exogenously in males with testicular hyperthermia or exposed to environmental toxicity. To maintain the optimal functioning of sperm cells, it is, therefore, necessary to balance the redox potential, i.e., to balance ROS by antioxidants. The purpose of this review is to present the antioxidant defense systems in semen.

## Introduction

Molecular studies of the underlying causes of male infertility have revealed a significant share of oxidative stress (OS), which is defined as an imbalance in the redox state of the body caused either by too high levels of oxidants or, conversely, by too little antioxidants. When excessive amounts of reactive oxygen species (ROS) are produced or antioxidant activity fails, the equilibrium between oxidation and reduction is disrupted, causing oxidative stress (OS). Sperm are especially sensitive to OS. They contain very low levels of enzymatic antioxidants which are insufficient to protect sperm from high levels of ROS, and the cytoplasm has only small concentrations of the enzyme capable of neutralizing them. A growing body of evidence suggests that the imbalance between oxidants and antioxidants in semen leads to metabolic and functional disorders of male reproductive cells and may be the main cause of some types of infertility [1]. The oxygen metabolism of human sperm produces various potentially harmful ROS on the plasma membrane of sperm with a high content of polyunsaturated fatty acids [2, 3]. ROS are divided into oxygen radicals (hydroxyl radical, nitric oxide radical, and superoxide anion radical) and nonradical derivatives (hydrogen peroxide, peroxynitrite anion, and hypochlorous acid) [4]. Sperm produce them in very small amounts, providing beneficial functional effects, including the initiation of sperm capacitation, regulation of sperm maturation, and enhancement of cell signaling pathways [5]. Moreover, ROS are involved in the condensation of sperm chromatin, regulating the number of reproductive cells by inducing apoptosis or proliferation of spermatogonia [6]. On the other hand, high levels of ROS may have an adverse effect on sperm function through the initiation of DNA damage, lipid peroxidation, loss of membrane integrity, increased membrane permeability, inactivation of cellular enzymes, and cell apoptosis [7–9] ultimately leading to infertility [10]. DNA damage caused by oxidation breaks the chromosome, leading to mutations [11]. Single strand lesions, and in particular DNA double strand breaks, are the most genotoxic and irreparable lesions [12]. It has been shown that sperm DNA integrity may be irreversibly compromised in response to increased susceptibility to OS [13]. Several studies have confirmed the important role of intact sperm DNA in normal embryonic development [14–16]. According to Virro et al. [17], moreover, it has been shown that changes in the paternal genome can influence the development of embryos even with direct injection of semen into the oocyte via ICSI. Other researchers have found that higher levels of DNA fragmentation in sperm are associated with lower rates of blastocyst formation [17]. Nasr-Esfahani et al. [18] showed that sperm-derived embryos with high levels of DNA damage are less likely to reach later developmental stages or blastocysts. Moreover, the fragmentation of sperm DNA showed a negative correlation with the rates of blastulation and pregnancy even in the case of good quality oocytes, and high levels of DNA damage favored the arrest of embryo development [19].

We distinguish at least three main sources of ROS in semen: the membrane NADPH oxidase, an enzyme complex contained in the cell membrane, and the others are mitochondria. It was also found that the production of superoxide anions involves NADH oxidoreductase (diaphorase), an enzyme located in the middle part of the sperm, which is closely related to the mitochondrial chain and xanthine oxidase present in both semen and semen plasma [20]. The third source is leukocytes, which under physiological conditions produce up to 1000 times more ROS than sperm [21]. It is worth emphasizing that sperm with abnormal morphology (mainly with cytoplasmic residues indicating their immaturity and reduced fertility potential) produce greater amounts of ROS than sperm with normal structure [22, 23]. There is also a difference in the amount of ROS produced by sperm at different stages of maturation. Apart from endogenous factors, such as immature sperm, leukocytes, and varicocele, potential causes can also be sought exogenously in males with testicular hyperthermia or those exposed to environmental toxicity.

To maintain the optimal functioning of sperm cells, it is, therefore, necessary to balance the redox potential, i.e., to balance ROS by antioxidants [24]. Seminal fluid is rich in them and at the same time performs nutritional and protective functions for sperm. Antioxidants can be divided into two forms: enzymatic and non-enzymatic antioxidant systems [25]. The enzyme system consists of glutathione peroxidase, superoxide dismutase, and catalase. These enzymes occur naturally in sperm or semen plasma and are thought to come from the prostate. On the other hand, the non-enzymatic system consists of many compounds that are delivered to the body with food (including dietary supplements).

The purpose of this review is to present the antioxidant defense systems in semen.

## Antioxidant Enzymes

The major system of antioxidant enzymes in semen is called the enzyme triad of superoxide dismutase, catalase, and glutathione peroxidase.

Catalysts for the dismutation of superoxide anions include superoxide dismutases or superoxide oxidoreductases. Two forms of their intra- and extracellular occurrence have been identified. Two of the intracellular forms are copper-zinc SOD, which is located mainly in the cytoplasm and contains, as the name suggests, the elements copper and zinc (Cu, ZnSOD, SOD-1) in the active center and SOD manganese, which is mainly located in the mitochondrial matrix (MnSOD, SOD-2) with manganese in the active center. The extracellular form of SOD (EC-SOD, SOD-3) located in the extracellular space is structurally similar to SOD-2, but instead of manganese, it has zinc and copper in its active center [20, 26]. SOD is highly active in semen plasma, with 75% of its activity related to SOD-1 activity and the remaining 25% to SOD-3. It has been shown that these two isoenzymes probably originate from the prostate [27].

Catalase (CT) in turn catalyzes the decomposition of hydrogen peroxide into molecular oxygen and water. Characteristic for CT is the heme system with a centrally located iron atom. Its activity has been demonstrated in peroxisomes, mitochondria, endoplasmic reticulum, and cytosol in various cells [28]. In the ejaculate, it has been located in human and rodent sperm, and in semen plasma, the source of which is the prostate [20]. Catalase is involved in the activation of sperm capacitation induced by nitric oxide, which is a complicated mechanism of hydrogen peroxide [29].

The enzyme catalyzing the reduction of hydrogen peroxide and organic peroxides, including peroxides of phospholipids in the antioxidant system in semen, is glutathione peroxidase (GPX) [30]. In its active place, it contains selenium in the form of selenocysteine. In sperm, it is located mainly in the mitochondrial matrix [27], but a nuclear form has also been found, which has a protective effect on sperm DNA against OS damage and is actively involved in the chromatin condensation process [31]. The presence of GPX was also demonstrated in semen plasma, which suggests its origin also from the prostate, as well as the enzymes mentioned above [32].

The complex spatial structure of enzyme molecules determines both the catalytic capabilities and the sensitivity to many environmental factors. The basic mechanism of enzyme action is related to the spatial adjustment of the substrate to the catalytic center of the enzyme.

## Non-enzymatic Antioxidants

There are also non-enzymatic antioxidants in the cell, which also play a key role in protecting sperm cells from OS. Table 1 shows the main antioxidants belonging to the non-enzyme and enzyme groups, along with their location and function.

**Table 1:** Location and function of major non-enzymatic and enzymatic antioxidants

Antioxidants	Locations	Function	References
Glutathione	Non-protein thiol in cell	Deactivates free radicals	[33–35]
Carotenoids	Lipid soluble antioxidant in membrane tissue	Maintains cell membrane integrity; regulates spermatogenesis and proliferation of epithelial cells	[36]
Vitamin E (α–tocopherol)	Mostly in membranes	Detoxifies products of membrane lipid peroxidation (LPO)	[37, 38]
Vitamin C (ascorbic acid)	Cytosol, chloroplast, mitochondria, peroxisome, vacuole	Detoxifies H_2_O_2_.	[30]
SOD	All eukaryotic cells	Lowers the steady-state level of O^2-^	[20, 26, 27]
CT	Intracellular organelles, peroxisomes	Lowers the level of H_2_ O_2_	[28, 29]
GPX	Cytoplasm, mitochondrial matrix	Lowers the level of H_2_ O_2_	[31, 32]

The group of non-enzymatic antioxidants also includes coenzyme Q10, which is especially present in high concentrations in the mitochondria of sperm involved in cell respiration and plays an integral role in energy production [39]. This positive effect promotes its use as a motility stimulant and antioxidant molecule. Interestingly, CoQ10 inhibits the formation of superoxide, providing protection against OS-induced sperm dysfunction. There was a significant negative correlation between CoQ10 levels and hydrogen peroxide, and a linear correlation was found between CoQ10 levels with the sperm count in the semen and their motility [40].

Selenium shows the ability to protect sperm DNA against OS damage in a mechanism that has not yet been well understood. Since selenium is the main component of selenoenzymes, its antioxidant properties are thought to stem from its ability to augment the function of glutathione. More than 25 selenoproteins exist, such as phospholipid hydroperoxide glutathione peroxidase (PHGPX) [41] and sperm capsular selenoprotein glutathione peroxidase [42], which help maintain sperm structural integrity [43]. Selenium deficiency has been most commonly associated with morphological sperm midpiece abnormalities and impairment of sperm motility [44].

The action of all antioxidants in the body ultimately comes down to counteracting the effects of oxidation processes, whether directly by catalyzing enzymatic reactions or indirectly as compounds that deactivate free radicals and stop the cascade of chain reactions. The antioxidant systems undoubtedly also include groups of compounds participating in the constant restoration of the antioxidant potential, because all molecules participating in the reactions are consumed and must be restored [27].

Due to the lack of part of the cytoplasm, mammalian sperm cells have a reduced ability of antioxidant defense, which makes them less resistant to oxidative damage [45]. Thus, the presence of antioxidant enzymes in semen is extremely important to prevent the negative effects of OS during sperm storage. The addition of natural antioxidants to dilute the semen has become necessary due to the lack of sufficient natural antioxidant reserves to maintain the satisfactory biological potential of the sperm. Plant-derived and synthetic compounds are widely used in sperm preservation technology of various species and are currently considered a valuable source of antioxidants, mainly due to the lack or low incidence of side effects [46–50].

## Methods of Supporting the Natural Antioxidant System

Antioxidants are widely available compounds that are commonly used in the treatment of male infertility. The main division of methods focuses on oral therapy and sperm supplementation. Many studies report a beneficial effect of antioxidant therapy on the basic biological parameters of semen, advanced sperm function, the results of assisted reproductive therapy, and the live birth rate [51, 52]. The most commonly tested antioxidants and their doses were as follows: α-tocopherol (400 mg), ascorbic acid (500–1000 mg), carnitine (500–1000 mg), N-acetyl cysteine ​​(NAC; 600 mg), coenzyme Q10 (CoQ10, 100–300 mg) zinc (Zn) (25–400 mg), selenium (Se) (200 μg), folic acid (0.5 mg), and lycopene (6–8 mg) [81–86].

### α-Tocopherol (Vit. E)

α-Tocopherol (Vit. E) is a fat soluble organic compound found mainly in cell membranes. It neutralizes hydroxyl free radicals and superoxide anions, effectively inhibiting lipid peroxidation initiated by ROS at the level of plasma membranes. A direct relationship was found between the level of vitamin E in the semen plasma and the percentage of motile sperm in the ejaculate [53]. Moreover, Omu et al. [54] showed that infertile men sperm with significantly lower levels of vitamin E. Moreover, patients treated with vitamin E for 6 months had a much lower percentage of sperm with LPO; additionally, vitamin E may increase the pregnancy rate in cases of asthenozoospermia [55]. It has also been shown that the simultaneous administration of α-tocopherol and selenium increases sperm motility in infertile men [56, 57]. Recent studies have shown a protective effect of vitamin E on sperm motility and oxidative stress in rats treated with valproic acid [58].

### Ascorbic Acid (Vit. C)

The concentration of this compound is almost ten times higher in the semen plasma than in the blood serum [59, 80]. It neutralizes hydroxyl, superoxide, and peroxide radicals, protecting against endogenous oxidative damage [60, 80]. It has been shown that the addition of vitamin C (and vitamin E) to the semen of men with normozoospermia and asthenozoospermia reduces the degree of DNA fragmentation caused by ROS [61, 62]. In turn, Akmal et al. [63] achieved an improvement in total sperm count, motility, and morphology after the administration of vitamin C in the dose of 2000 mg/day.

### Carnitines [L-Carnitine (LC) and L-Acetyl Carnitine (Lac)]

Carnitine is involved in the sperm maturation process in the epididymis. The concentration of free L-carnitine in the tail of the epididymis is 2,000 times higher than in the blood plasma. Carnitine is taken from the blood and then transferred to the epididymis through active epithelial pumps that are stimulated by androgens [87, 88]. Its deficiency may lead to decreased sperm motility [89]. Many in vitro studies of sperm cultured in media containing carnitine have reported an increase in cell mobility and viability compared to controls [64, 65]. The effectiveness of L-carnitine as an antioxidant has been observed both in humans [66] and in rats [67, 68]. More recently, Abd-Elrazek and Ahmed-Farid [69] showed that administration of L-carnitine to adult oligospermic rats attenuated the cytotoxic effect of busulfan by improving sperm parameters, reducing oxidative stress, and maintaining cellular energy. Moreover, Nazari et al. [70] proved that antioxidant supplementation containing 1500 mg of L-carnitine can improve sperm quality in infertile men by improving cell concentration and overall motility.

### N-Acetylcysteine

An amino acid converted into cysteine in tissues, a precursor to glutathione, is a compound capable of direct reduction of OS by scavenging hypochlorous acid and hydroxyl radicals [71]. Numerous studies (in vitro) showed a significant decrease in the level of ROS generation and an improvement in the motility of sperm incubated in the presence of NAC [72]. In turn, Jannatifar et al. [73] proved that NAC therapy increases the number and percentage of motile sperm, while reducing the number of sperm cells with abnormal morphology and damaged DNA. In turn, in adjunctive therapy after varicocelectomy, as reported by Barekata et al. [74], NAC may have a positive effect on chromatin integrity and pregnancy rate.

### Zinc

Zinc plays an essential role in RNA and DNA metabolism, signal transduction, gene expression, and regulation of apoptosis [80]. Moreover, it has been shown that zinc has a significant protective effect on the structure of sperm. The use of zinc in asthenozoospermic patients reduces oxidative stress, apoptosis, and DNA fragmentation of their sperm [41, 75]. Recent studies have shown that supplementation of sperm media with zinc protects bull sperm against exogenous oxidative stress and improves their ability to support embryo development [76]. Moreover, other researchers [77] reported that supplementing zinc oxide nanoparticles in the cryopreservation medium minimizes the freeze-thaw-induced damage to spermatozoa.

### Lycopene

Lycopene is a naturally synthesized carotenoid is found in fruits and vegetables. Its powerful ROS-extinguishing abilities make it a major contributor to the human redox defense system [78, 80]. The results of studies by Tvrda et al. [79] suggest that lycopene exhibits significant ROS-scavenging and antioxidant properties, which may prevent sperm lesions caused by oxidative stress and preserve the functionality of male reproductive cells.

## Limitations Related to ROS Balancing in Reproductive Medicine

It has been a long time since the link between redox homeostasis and impaired male fertility was established. The numerous antioxidant molecules present in the male reproductive system maintain the physiological level of ROS, which allows them to stimulate almost the entire process pathway from steridogenesis to oocyte fertilization.

Undoubtedly, the greatest challenge of reproductive medicine is the prevention of ROS-induced reproductive disorders, as well as the restoration of redox homeostasis when reproductive abnormalities are detected. Hence, antioxidant therapies represent a potential preventive and therapeutic alternative in the management of patients at risk of ROS imbalance.

Various antioxidants can have a synergistic effect on the removal of active oxygen, which was confirmed in the studies by Ren et al. [90]. This may be due to the different binding rate to active ROS and the different mode of action. Moreover, the synergistic effect of two different antioxidants has the potential to improve selected parameters of the preserved semen compared to adding only one antioxidant to the extender.

Unfortunately, there is still a lack of research providing information on the potential mechanism of antioxidant action, which delays the development of more effective and broader antioxidant therapies. Therefore, it is necessary to conduct further research in this direction to be able to determine the dosage of antioxidant supplementation, which is currently not regulated. It should be remembered that unregulated (improper) supplementation can also lead to pathological abundance of antioxidants, also causing redox dysregulation and inhibiting important reproductive processes.

Finally, a significant problem with the use of oxidative therapies is the difficulty in distinguishing between normal and abnormal ROS concentrations, which makes it difficult to accept the ROS measurement as a standard process in routine semen analysis for clinical applications.

## Conclusion

The growing awareness of the factors of the pathogenesis of many diseases that the humanity of the twenty-first century has to deal with prompts us to look for mechanisms that will help to counteract these diseases and their consequences. Research in recent years has shown that oxidative stress, caused by reactive oxygen species and free radicals, can underlie the occurrence of many reproductive disorders and is directly related to infertility or complete sterility. Although the antioxidant defense system is active in semen, its activity is limited due to the low amount of sperm cytoplasm; therefore, scientists are still making efforts to search for substances that can support the natural antioxidant defense system of cells.

The therapeutic value of antioxidants depends on their ability to prevent or alleviate specific conditions. Despite their protective effect against oxidative stress, they exhibit certain features that ultimately limit their use. These limitations are their instability to oxidative processes or changes in pH, as well as the possibility of achieving concentrations that cause an antioxidative effect. Very often, these compounds show low solubility, which requires them to be administered with solvents (such as ethanol or dimethylsulfoxide) or with high concentrations of surfactants that can lead to toxicity.

To date, many compounds with antioxidant properties have been studied for the treatment of infertile men. Understanding the antioxidant mechanism of action of commonly used compounds is important before considering the evidence related to their use in clinical practice.

## Data Availability

Not applicable.

## Abbreviations

APX: Ascorbate peroxidase
CT: Catalase
ROS: Reactive oxygen species
OS: Oxidative stress
SOD: Superoxide oxidoreductases
GPX: Glutathione peroxidase
CoQ10: Coenzyme Q10
PHGPX: Phospholipid hydroperoxide glutathione peroxidase
NAC: N-acetyl cysteine
LPO: Lipid peroxidation
